# *Streptococcus sp*. in neonatal endotracheal tube biofilms is associated with ventilator-associated pneumonia and enhanced biofilm formation of *Pseudomonas aeruginosa* PAO1

**DOI:** 10.1038/s41598-017-03656-2

**Published:** 2017-06-13

**Authors:** Yun Pan, Sijie Song, Xiaoli Tang, Qing Ai, Danping Zhu, Zhenqiu Liu, Jialin Yu

**Affiliations:** 1 Department of Neonatology, Children’s Hospital of Chongqing Medical University, Chongqing, China; 2 Key Laboratory of Pediatrics in Chongqing, CSTC2009CA5002, Chongqing, China; 3 Ministry of Education Key Laboratory of Child Development and Disorders, Chongqing, China; 4 Chongqing International Science and Technology Cooperation Center for Child Development and Disorders, Chongqing, China

## Abstract

Ventilator-associated pneumonia (VAP) is a serious complication of mechanical ventilation leading to high morbidity and mortality among intubated neonates in neonatal intensive care units (NICUs). Endotracheal tube (ETT) biofilm flora were considered to be responsible for the occurrence of VAP as a reservoir of pathogens. However, regarding neonates with VAP, little is known about the complex microbial signatures in ETT biofilms. In the present study, a culture-independent approach based on next generation sequencing was performed as an initial survey to investigate the microbial communities in ETT biofilms of 49 intubated neonates with and without VAP. Our results revealed a far more complex microflora in ETT biofilms from intubated neonates compared to a previous culture-based study. The abundance of Streptococci in ETT biofilms was significantly related to the onset of VAP. By isolating Streptococci in ETT biofilms, we found that Streptococci enhanced biofilm formation of the common nosocomial pathogen *Pseudomonas aeruginosa* PAO1 and decreased IL-8 expression of airway epithelia cells exposed to the biofilm conditioned medium of PAO1. This study provides new insight into the pathogenesis of VAP among intubated neonates. More studies focusing on intubated neonates are warranted to develop strategies to address this important nosocomial disease in NICUs.

## Introduction

Ventilator-associated pneumonia (VAP) is a serious complication related to mechanical ventilation in neonatal intensive care units (NICUs), and it accounts for increased health care costs, prolonged hospital stays and hospital mortality^1^. It ranks as the second most common nosocomial infection in pediatric intensive care units^2^ and is the leading cause of death among nosocomial infections in ventilated patients^3, 4^. The burden of VAP in NICUs is much more serious in developing countries than in developed countries^1, 5^. According to a recent meta-analysis conducted in China, the incidence and case fatality rates of VAP in NICUs were 42.8% and 16.4%, respectively^5^. Therefore, VAP poses a major challenge to public health and has considerable health and economic consequences in China.

During ventilation, the endotracheal tube (ETT) provides a direct conduit for pathogenic bacteria to enter the normally sterile lower respiratory tract and lung parenchyma^6^. Remarkably, bacteria can adhere and form a biofilm on the surface of the ETT soon after intubation^6–8^. These biofilms are complex communities of microbes that produce a matrix of hydrated extracellular polymeric substances that mainly include polysaccharides, proteins, nucleic acids and lipids^9^. Biofilm cells can continuously access the lower airway through ventilator gas flow and aspiration^8^. Mounting evidence shows that biofilms on ETTs serve as significant and persistent reservoirs of pathogens to cause VAP^7, 10, 11^. Strategies involving modified ETTs to prevent or remove ETT biofilms were proven to reduce VAP occurrence in adults, including cuffed ETTs and silver or other nanoparticle coated ETTs^12, 13^. However, for intubated neonates, the CDC suggests that cuffed ETTs are not available and the safety of nanoparticle coated ETTs for neonates is uncertain^1, 12^; other strategies, therefore, are warranted to address ETT biofilms among ventilated neonates.

In addition to the ETT shape and material, bacterial factors should also be highlighted, considering that biofilms are generated by bacteria. The biofilm formation ability differs between bacterial species and is influenced by bacterial members both synergistically and antagonistically^14^. Therefore, identifying complex microbial members in biofilms is essential; yet, studies on biofilm bacterial populations among intubated neonates are limited in the literature. A previous study conducted among 29 intubated neonates demonstrated the predominance of *Staphylococcus* species and normal flora, such as *Streptococcus viridans*, in ETT biofilms based on a culture-dependent approach^15^. However, the culture-dependent approach showed a significant culturing bias due to the failure to recover and grow all of the bacteria in the ETT biofilm^16^. Culture-independent approaches are available to identify microorganisms, including fastidious or nonculturable organisms in ETT biofilms, on the basis of variations in the domains of the 16S ribosomal RNA (rRNA) gene^16–18^. For example, recent studies among adults undergoing mechanical ventilation demonstrated a much higher diversity of microflora in ETT biofilms via the culture-independent method compared with the culture-dependent method^16–18^. However, similar studies among intubated neonates are scarce.

To fill in these knowledge gaps described above, we conducted the present study using ETT samples collected from intubated neonates, and the patient data available at Children’s Hospital of Chongqing Medical University (CHCMU), China. The NICU in CHCMU is a 50-bed, level 3 NICU and serves as the referral base for the emergency medical treatment of critically ill and premature newborns from Chongqing. Using the culture-independent method based on Illumina Miseq platforms, we aimed to present a global view of the microbial communities in ETT biofilms from ventilated neonates with and without VAP. To further characterize the pathogenesis of the ETT biofilm flora in VAP, representative bacterial species were isolated from ETT biofilms of VAP patients, and their biofilm formation capacities were assessed *in vitro*. With a better understanding of the ETT biofilm flora among intubated neonates, corresponding prevention and treatment strategies can be developed to decrease the incidence of VAP among neonates.

## Results

### Study patients

Neonates with sustained mechanical ventilation for more than 48 hours in CHCMU during the study period from January 31, 2014, to July 31, 2014, were considered in the study. Considering that the underlying disease prior to intubation may influence the microbiota in ETT biofilms, patients diagnosed with neonatal respiratory distress syndrome (NRDS) and pneumonia during the study period were consecutively involved and classified into NRDS and pneumonia groups. During the study period, we collected the data of 62 intubated neonates. However, the study included all of the available 49 (79.0%) patients, while the other 13 (21.0%) were excluded from the study due to sample contamination (n = 5) and PCR failure (n = 8). The five contaminated samples were polluted in the process of sample procession by non-sterile solutions due to improper operations. Considering the potential selection bias due to the 13 excluded cases, the distribution of the basic demographic (sex, gestational age and birth weight) and clinical characteristics (intubation duration, VAP or no VAP) between the 49 study patients and the 13 missing cases were compared (Table 1). No significant difference was identified in the distribution of the included demographic and clinical characteristics (*P* > 0.05, Table 1). Of the 49 study patients, 23 were diagnosed with NRDS, including 17 VAP cases; the remaining 26 patients were pneumonia cases, including 13 VAP cases. The baseline characteristics between VAP and non-VAP patients were compared among the NRDS and pneumonia groups (Tables 2 and 3). The included baseline characteristics were similarly distributed between patients with and without VAP, except for the intubation duration. The intubation duration of VAP cases was significantly longer than those without VAP among NRDS patients (*P* < 0.05, Table 2).

**Table 1:** Comparison of baseline characteristics between 49 study patients included in the study and 13 patients who were not included in the study.

Characteristics	Included cases (N = 49)	Not included cases (N = 13)	*P* value
Sex (male)	33	9	1.00^a^
Gestational age (x ± s, week)	34.3 ± 4.1	34.6 ± 3.8	0.69^b^
Birth weight (x ± s, kg)	2.2 ± 0.8	2.5 ± 1.1	0.23^b^
Total intubation duration [P50 (P25–P75), day]	4.7 (3.0–5.6)	5.0 (4.1–8.2)	0.3^c^
VAP	30	5	0.14^d^

^a^Based on Fisher’s exact test.

^b^Based on unpaired Student’s t-test.

^c^Based on Mann–Whitney U test.

^d^Based on Chi-square test.

**Table 2:** Comparison of demographic and clinical characteristics between non-VAP and VAP infants among neonates with NRDS in the study.

Characteristics	Non-VAP (N = 6)	VAP (N = 17)	*P* value
Gender (male)	4	10	1.00^a^
Gestational age (x ± s, week)	33.4 ± 3.5	32 ± 2.3	0.26^b^
Birth weight (x ± s, kg)	1.9 ± 0.7	1.8 ± 0.5	0.58^b^
Total intubation duration [P50 (P25–P75), day]	3.6 (2.2–6.1)	5.3 (4.6–10.9)	0.05^c^
Antibiotics	Cefoxitin	Moxalactam, Piperacillin/tazobactam Cefoperazone/sulbactam, Panipenem/betamipron or Lmipenem, etc.	

^a^Based on Fisher’s exact test.

^b^Based on unpaired Student’s t-test.

^c^Based on Mann–Whitney U test.

**Table 3:** Comparison of demographic and clinical characteristics between non-VAP and VAP infants among neonates with pneumonia in the study.

Characteristics	Non-VAP (N = 13)	VAP (N = 13)	*P* value
Gender (male)	9	10	1.00^a^
Gestational age (x ± s, week)	36.8 ± 4.5	36.0 ± 4.6	0.73^b^
Birth weight (x ± s, kg)	2.6 ± 1.2	2.5 ± 0.6	0.84^b^
Total intubation duration [P50 (P25–P75), day]	3.5 (2.8–5.0)	4.0 (2.5–5.0)	0.79^c^
Antibiotics	Piperacillin/tazobactam	Piperacillin/tazobactam, Cefoperazone/sulbactam, Panipenem/betamipron, or Lmipenem, etc.	

^a^Based on Fisher’s exact test.

^b^Based on unpaired Student’s t-test.

^c^Based on Mann–Whitney U test.

### Miseq sequencing and initial data processing

We obtained all 49 ETT samples collected from the 49 study patients at extubation. All of the study samples were successfully processed for sequencing. After denoising and chimera removal, a total of 787, 916 reads remained and were de-multiplexed. The mean number of reads was 14, 591 per sample. The high coverage coefficients of sequencing (over 99.9%) showed that the magnitude of the sequencing was sufficient to capture the operational taxonomy unit (OTU) diversity in all of the study samples.

### Microbial diversity in ETT biofilms

The Shannon-Wiener index in the ETT biofilm flora among pneumonia patients was significant higher than in those among NRDS subjects (3.36 ± 1.51 vs. 0.84 ± 0.18, *P* < 0.05, Table 4). Similar results were exhibited in the comparisons of the Ace and Chao index between pneumonia and NRDS patients (*P* < 0.05, Table 4). The Simpson’s index in pneumonia patients was significantly lower than that in NRDS subjects (*P* < 0.05, Table 4). All four biodiversity indexes above indicated a lower richness and diversity of microflora in the ETT biofilms of NRDS patients than of pneumonia patients.

**Table 4:** Comparison of microbial diversities of endotracheal tube biofilm flora of the study samples achieved from NRDS and pneumonia patients in the study.

	NRDS (N = 23)	Pneumonia (N = 26)	*P* value
Shannon-Wiener index	0.84 ± 0.18	3.36 ± 1.51	<0.01^a^
Simpson’s index	0.47 ± 0.06	0.19 ± 2.22	<0.01^a^
Ace index [P50 (P25–P75)]	22 (17–32)	334 (198–356)	<0.01^b^
Chao index	20.52 ± 10.37	286.04 ± 105.27	<0.01^a^

^a^Based on adjusted t-test.

^b^Based on Mann–Whitney U test.

**Figure 1:**
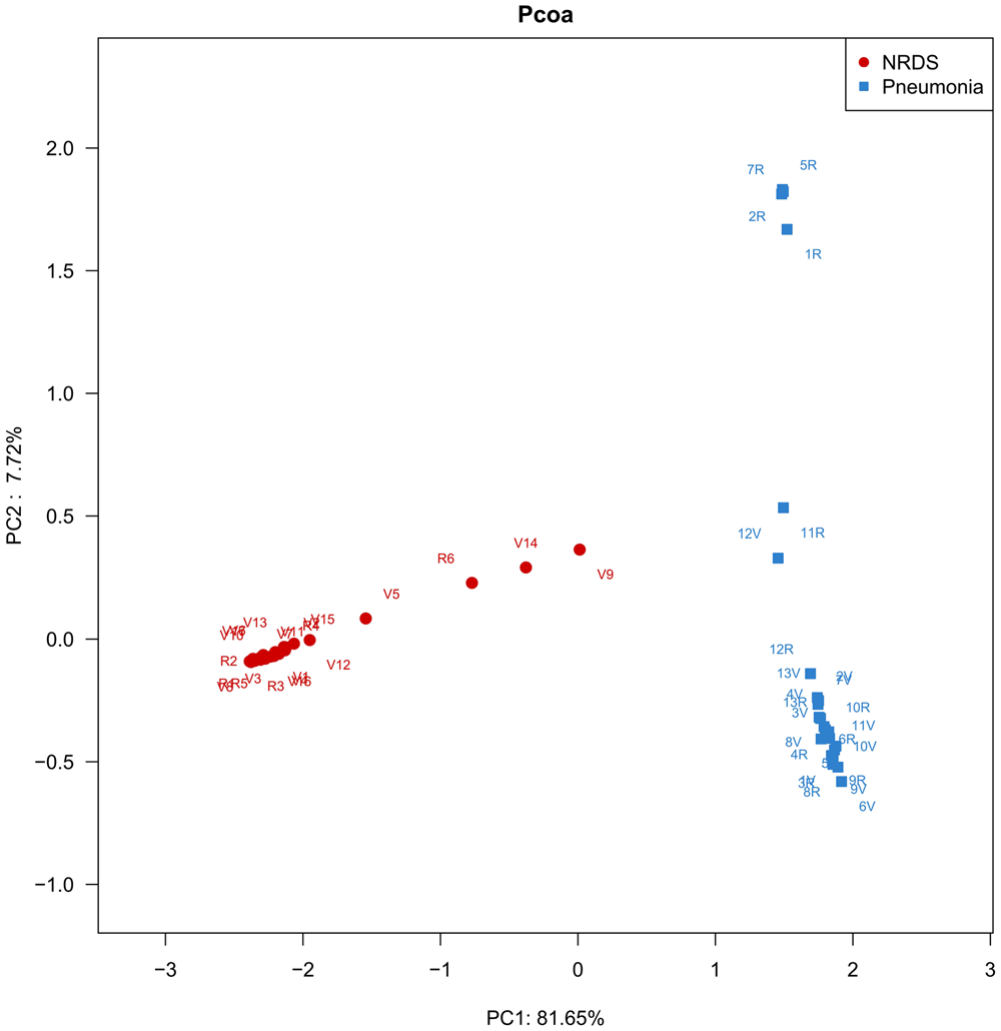
Principal coordinate analysis (PCoA) of the study samples based on the Bray-Curtis distance of bacteria communities. Samples collected from NRDS patients are represented by circles (red), including 17 with (V1–V17) and 6 without (R1–R6) ventilator-associated pneumonia (VAP) cases. Samples collected from pneumonia patients are represented by squares (blue), including 13 with (1V–13V) and 13 without (1R–13R) ventilator-associated pneumonia (VAP) cases.

Principal coordinates analysis (PCoA) to assess β diversity was also conducted between the NRDS and pneumonia subjects. PCoA was used to visualize the effects of the two major factors on the community composition. The distribution of the pneumonia samples was much more dispersed compared with the NRDS samples (Fig. 1). Additionally, the pneumonia samples were clearly separated from the NRDS samples, indicating the distinct bacterial patterns between pneumonia and NRDS subjects (Fig. 1). Adonis, one of the non-parametric multivariate statistical tests, also revealed that the microbial community structure was significantly different between the pneumonia and NRDS samples (F = 27.962, *P* = 0.001).

### Bacterial communities in ETT biofilms

**Figure 2:**
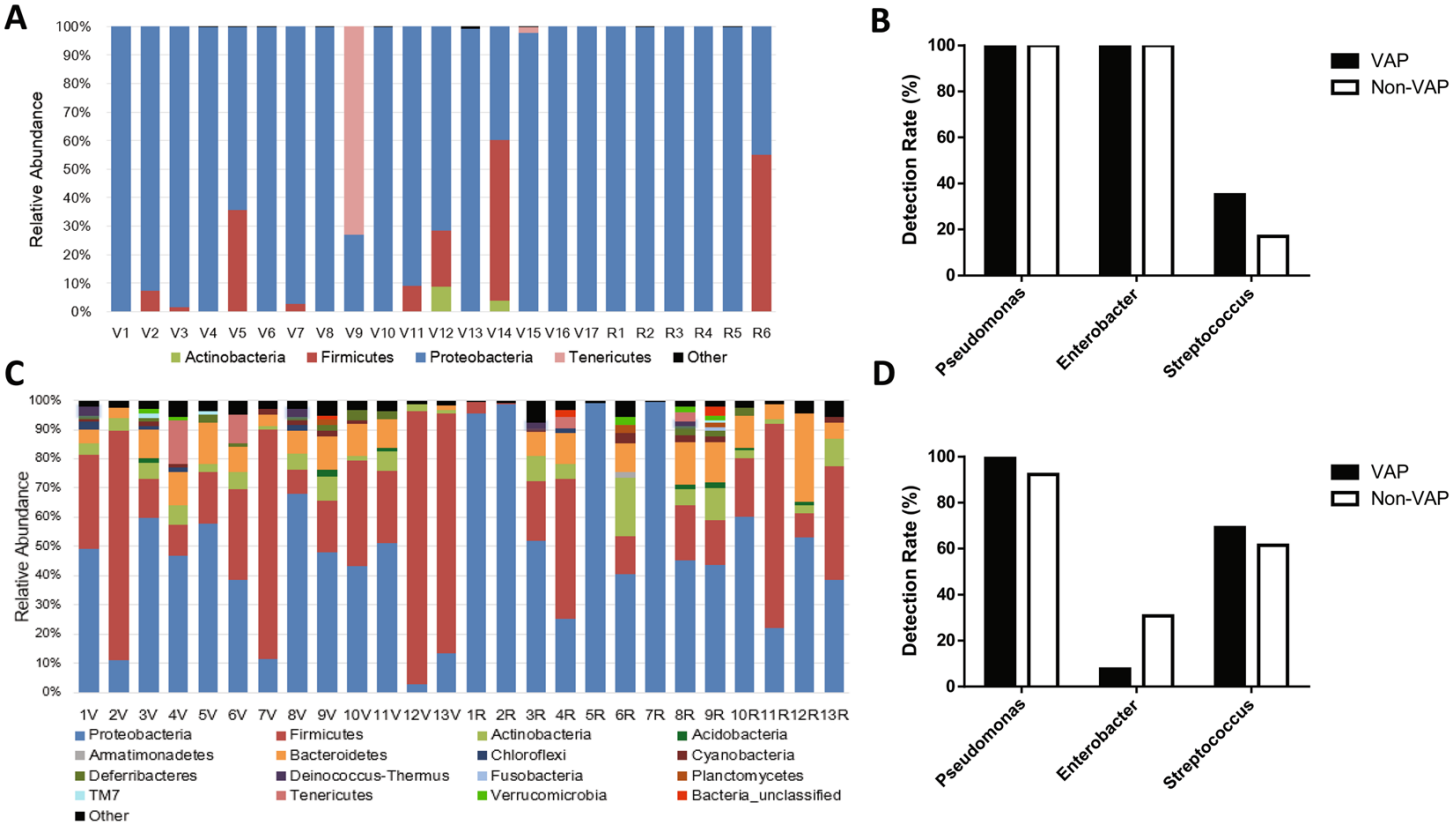
Bacterial communities in endotracheal tube biofilm. (**A**) Relative abundance of sequences belonging to different bacterial phyla in endotracheal tube biofilms of the 23 neonatal respiratory distress syndrome (NRDS) patients in the study. The y-axis indicates the relative abundance of phyla, and the x-axis indicates different samples from patients with concurrent ventilator-associated pneumonia (VAP) (V1–V17) and without VAP (R1–R6). (**B**) The detection rates of the three dominant genera in endotracheal tube biofilms of the 23 NRDS patients. (**C**) Relative abundance of sequences belonging to different bacterial phyla in endotracheal tube biofilms of the 26 patients with pneumonia in the study. The y-axis indicates the relative abundance of phyla, and the x-axis indicates different samples from patients with concurrent VAP (1V–13V) and without VAP (1R–13R). (**D**) The detection rates of the three dominant genera in endotracheal tube biofilms of the 26 pneumonia patients.

Regarding the distinct bacterial signatures in ETT flora between neonates with NRDS and those with pneumonia, the bacterial communities of the two groups were analysed separately. For NRDS patients, a total of 56 OTUs were identified in ETT biofilms, with an average of 17.7 OTUs per sample. *Proteobacteria* (87.4%, 306, 140/350, 330 reads) was dominant, followed by *Firmicutes* (8.7%, 30,324/350,330 reads) (Fig. 2A). At the genus level, *Pseudomonas* (44.7%, 156,626/350,330) and *Enterobacter* (42.3%, 148,357/350,330) were overrepresented, followed by *Streptococcus* (5.1%, 17,775/350,330). The detection rates of the three dominant genera in ETT biofilms among NRDS patients are shown in Fig. 2B. From the PCoA above (Fig. 1), no difference was identified between the global structures of microflora in the ETT biofilms of subjects with and without VAP. At the species level, over 99% (212,490/212,657) of reads identified as *Pseudomonas sp*. belonged to *Pseudomonas aeruginosa* PAO1. All of the reads identified as *Enterobacter sp*. and *Streptococcus sp*. failed to be further assigned at the species level.

Among pneumonia patients, 1,557 OTUs were identified in all of the ETT biofilm samples, with an average of 257.6 OTUs per sample. *Proteobacteria* (48.0%, 182,922/381,068) and *Firmicutes* (32.1%, 122,336/381,068) were the most widely distributed phyla among pneumonia patients (Fig. 2C). The PCoA already showed the different microbial patterns between NRDS and pneumonia patients, and to further confirm if the compositions of the ETT biofilm flora were different, we compared the abundance of *Proteobacteria* and *Firmicutes* (the two most overrepresented phyla in both groups) in the above two groups. We found significant differences in the ratio of the two phyla (*P* < 0.05). At the genus level, *Streptococcus* (19.4%, 73,772/381,068), *Pseudomonas* (12.0%, 45,798/381,068) and *Enterobacter* (6.4%, 24,433/381,068) were overrepresented. The detection rates of the three dominant genera in the ETT biofilms among pneumonia patients are shown in Fig. 2D. The PCoA showed no difference between the global microbial communities in the ETT biofilms of subjects with and without VAP (Fig. 1). Among the three dominant genera, 99.9% of the reads (73,691/73,772) assigned to *Streptococcus sp*. and all of the reads assigned to *Enterobacter sp*. failed to be further identified at the species level. Sixty-six percent of the reads identified as *Pseudomonas sp*. were assigned as *Pseudomonas aeruginosa* PAO1.

### Association between *Streptococcus sp*. in ETT biofilm and VAP

**Figure 3:**
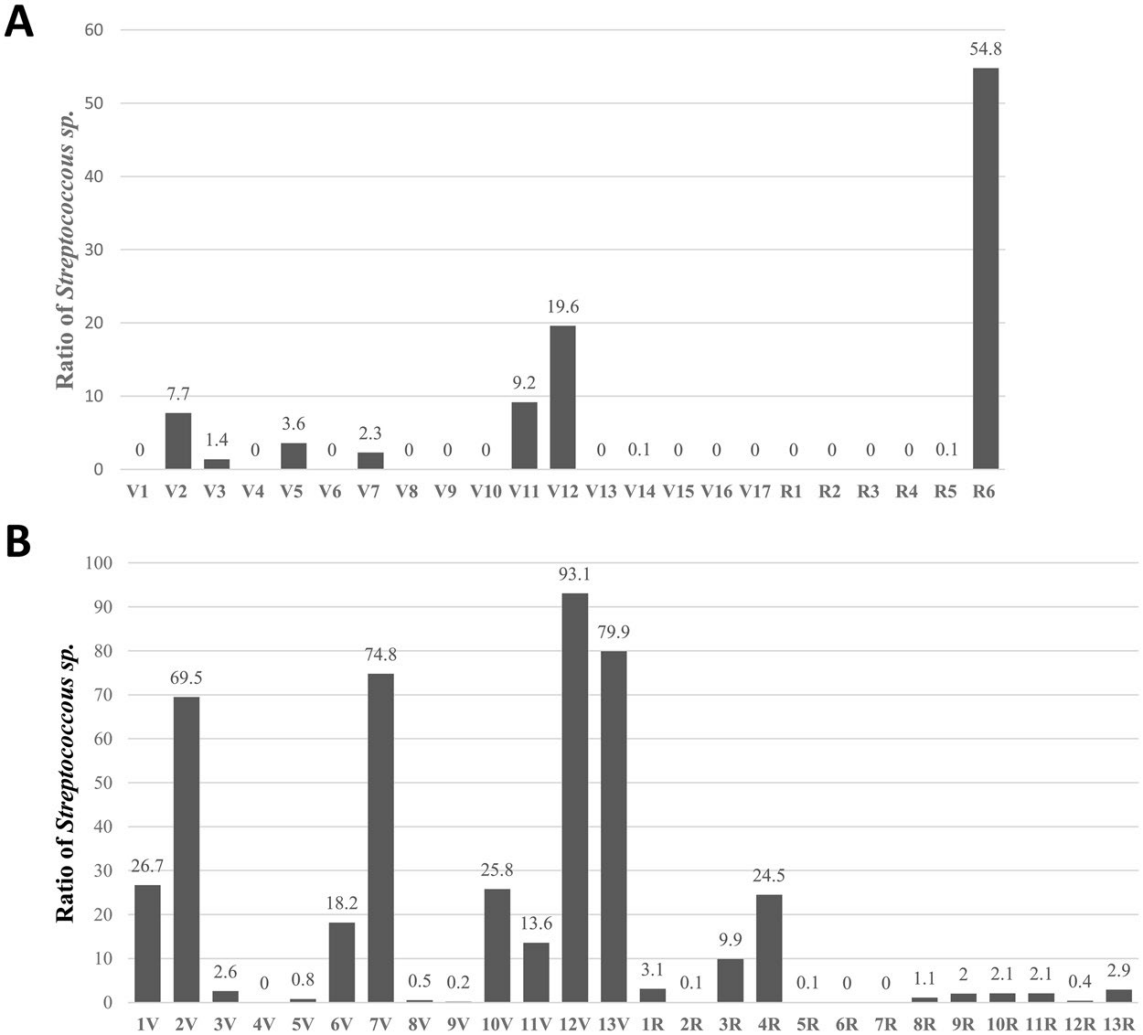
Ratio of *Streptococcus sp*. in endotracheal tube biofilm. (**A**) Relative abundance of sequences belonging to *Streptococcus sp*. in endotracheal tube biofilms of the 23 neonatal respiratory distress syndrome (NRDS) patients in the study. The y-axis indicates the relative abundance of *Streptococcus sp*. and the x-axis indicates different samples from patients with concurrent ventilator-associated pneumonia (VAP) (V1–V17) and without VAP (R1–R6) among the 23 NRDS patients in the study. (**B**) Relative abundance of sequences belonging to *Streptococcus sp*. in endotracheal tube biofilms of the 26 patients with pneumonia in the study. The y-axis indicates the relative abundance of *Streptococcus sp*., and the x-axis indicates different samples from patients with concurrent VAP (1V–13V) and without VAP (1R–13R) among the 26 patients with pneumonia in the study.

Considering the significantly distinct microbial signatures in ETT biofilms between NRDS and pneumonia subjects and their different proportions of VAP cases (17/23 vs. 13/26) in the study, the association between bacterial factors and VAP was analysed in NRDS and pneumonia patients. In NRDS patients, *Streptococcus sp*. OTUs were present (as at least 1% of sequencing reads) in ETT biofilms among eight patients, which included seven VAP patients and the remaining one non-VAP patient (Fig. 3A). However, the only non-VAP patient was diagnosed with culture-confirmed sepsis one week after extubation, which raised the concern that nosocomial infection may occur via mechanical ventilation. We further compared the demographic and clinical characteristics between VAP patients with (n = 6) and without *Streptococcus sp*. present (n = 11) in ETT biofilms. Although not statistically significant, the intubation duration was longer (13.0 [5.2–15.7] vs. 4.8 [4.2–10.4] days) and the white blood cell (WBC) counts were lower ([14.4 ± 5.1] × 10^9^/L vs. [22.8 ± 10.5] × 10^9^/L) in *Streptococcus sp*. present cases than the remaining *Streptococcus sp*. absent cases (*P* = 0.07 and 0.09, respectively, Table 5).

**Table 5:** Comparison of demographic and clinical characteristics between the *Streptococcus sp*. and the non-*Streptococcus sp*. groups among NRDS patients with VAP in the study.

Characteristics	*Streptococcus sp*. (N = 6)	Non-*Streptococcus sp*. (N = 11)	*P* value
Gender (male)	4	6	1.00^a^
Gestational age (x ± s, week)	31.9 ± 1.7	32.1 ± 2.6	0.89^b^
Birth weight (x ± s, kg)	1.5 ± 0.3	1.9 ± 0.6	0.21^b^
Total intubation duration [P50 (P25–P75), day]	13.0 (5.2–15.7)	4.8 (4.2–10.4)	0.07^c^
WBC count (*10^9^/L)	14.4 ± 5.1	22.8 ± 10.5	0.09^b^

^a^Based on Fisher’s exact test.

^b^Based on unpaired Student’s t-test.

^c^Based on Mann–Whitney U test.

In pneumonia patients, *Streptococcus sp*. were present in nine VAP patients and seven non-VAP patients (Fig. 3B). The Spearman test showed a significant correlation between the abundance of *Streptococcus sp*. in ETT biofilms and the onset of VAP (R = 0.49, *P* = 0.011 < 0.05). Although not statistically significant, we found that the WBC counts of VAP patients with *Streptococcus sp*. (n = 9) were lower than the remaining (n = 4) VAP cases ([12.1 ± 1.9] × 10^9^/L vs. [24.2 ± 8.8] × 10^9^/L) (*P* = 0.07, Table 6).

**Table 6:** Comparison of demographic and clinical characteristics between the *Streptococcus sp*. and the non-*Streptococcus sp*. groups among pneumonia patients with VAP in the study.

Characteristics	*Streptococcus sp*. (N = 9)	Non-*Streptococcus sp*. (N = 4)	*P* value
Gender (male)	6	4	0.50^a^
Gestational age (x ± s, week)	37.0 ± 4.5	34.5 ± 4.8	0.41^b^
Birth weight (x ± s, kg)	2.6 ± 0.6	2.3 ± 0.6	0.46^b^
Total intubation duration [P50 (P25–P75), day]	4.0 (2.5–5.0)	3.0 (1.9–5.3)	0.57^c^
WBC count (*10^9^/L)	12.1 ± 1.9	24.2 ± 8.8	0.07^d^

^a^Based on Fisher’s exact test.

^b^Based on unpaired Student’s t-test.

^c^Based on Mann–Whitney U test.

^d^Based on adjusted t-test.

### Identification of *Streptococcus sp*. in ETT biofilms

**Figure 4:**
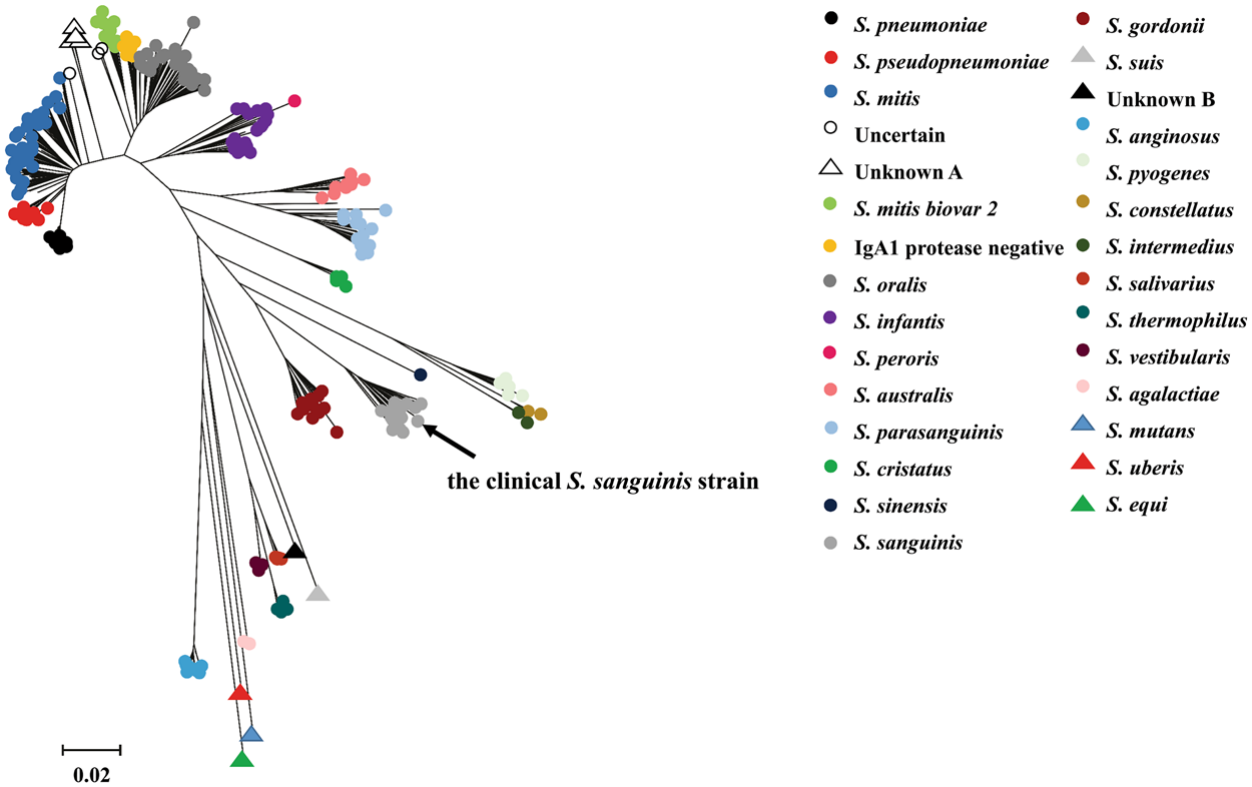
Phylogenetic tree of the clinical *S*. *sanguinis* strain.

In all of the 15 ETT biofilm samples in which *Streptococcus* was present among VAP patients, *Streptococcus pneumonia* (*S*. *pneumonia*) was not detected according to *S*. *pneumonia* specific PCR. Based on biofilm cultivation, only one *Streptococcus sp*. strain has been isolated. After performing multilocus sequence analysis (MLSA) of the seven housekeeping genes, the strain can be assigned to *Streptococcus sanguinis* (*S*. *sanguinis*) according to its phylogenetic tree with those established type stains (Fig. 4).

### Biofilm formation of the clinical *S*. *sanguinis* isolate

**Figure 5:**
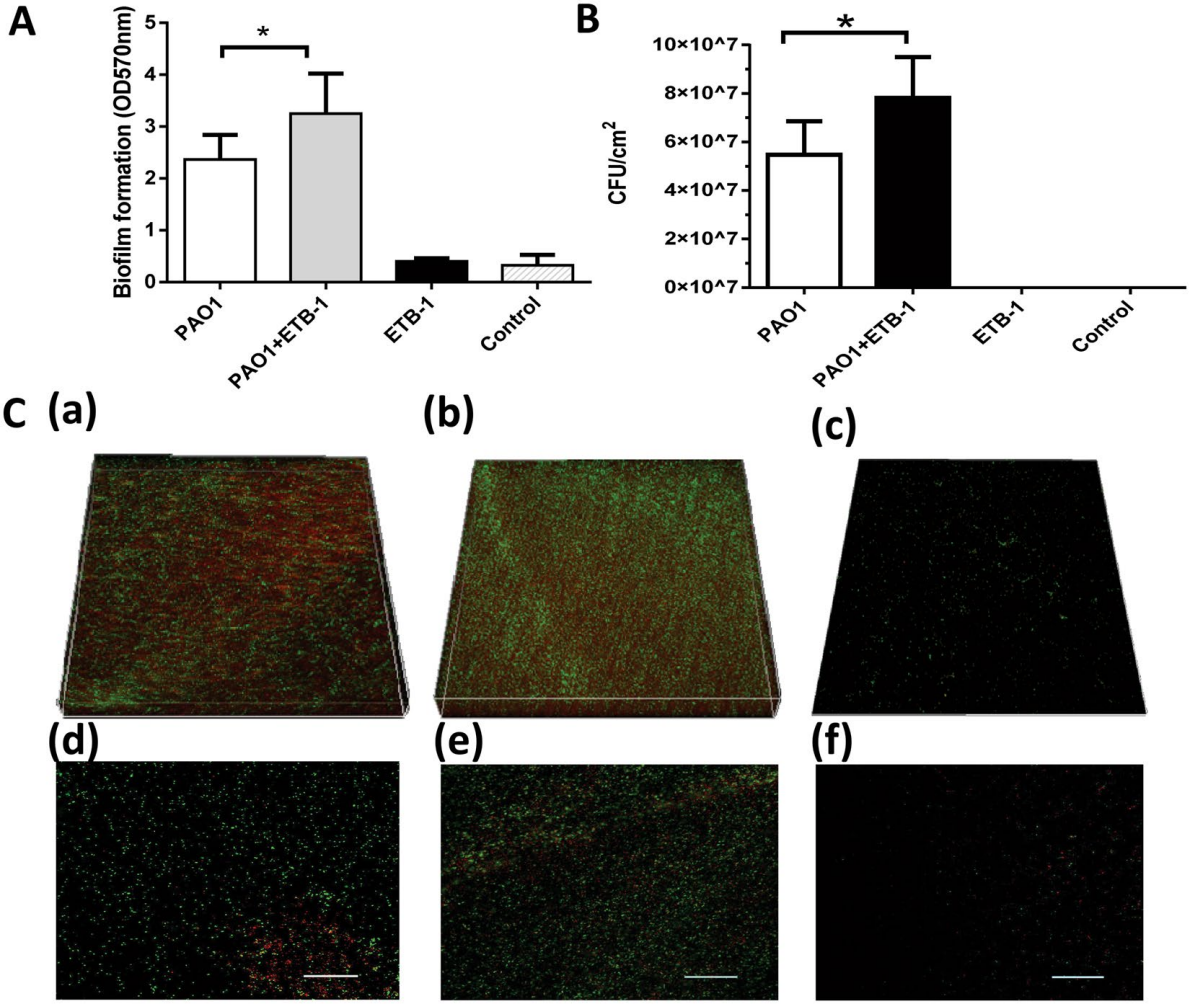
Biofilm formation assessed by crystal violet assay (**A**), plate count (**B**) and confocal laser scanning microscopy (CLSM) (×400) (**C**). PAO1 represents the 3-day *Pseudomonas aeruginosa* PAO1 biofilm. PAO1 + ETB-1 represents the 3-day *Pseudomonas aeruginosa* PAO1 and the clinical *S*. *sanguinis* strain co-cultured biofilm. ETB-1 represents the 3-day clinical *S*. *sanguinis* strain biofilm. *Indicates a significant difference between the PAO1 and the PAO1 + ETB-1 group (*P* < 0.05). Control represents the brain heart infusion medium applied in the study for biofilm culture. (**C**) Representative confocal laser scanning micrographs of biofilm in PAO1 (a,d), PAO1 + ETB-1 (b,e) and ETB-1 (c,f); the scale bars = 50 μm.

**Figure 6:**
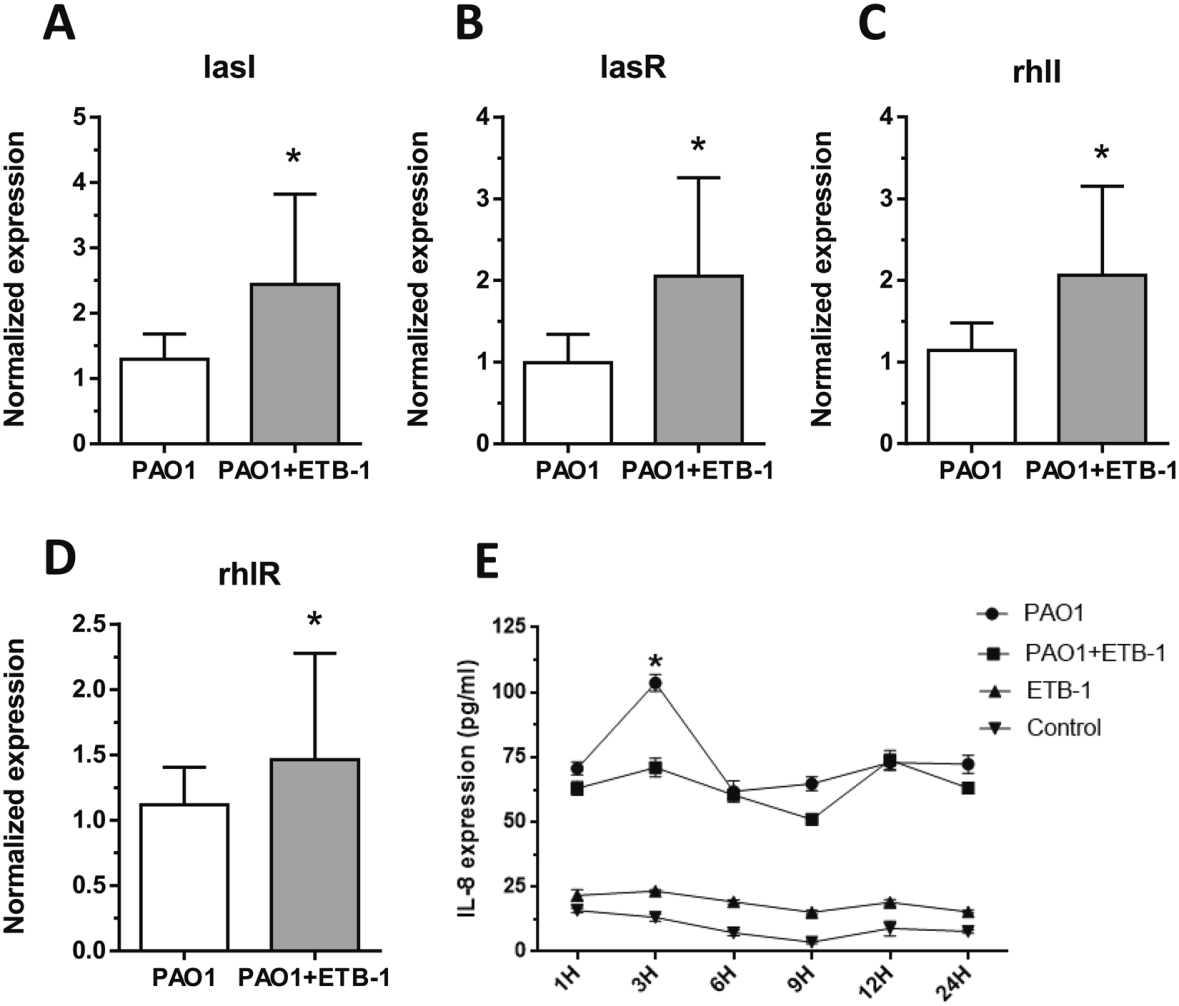
(**A**,**B**,**C**,**D**) Expressions of *Pseudomonas aeruginosa* Quorum sensing genes *lasI*, *lasR*, *rhlI* and *rhlR*. PAO1 represents the 3-day *Pseudomonas aeruginosa* PAO1 biofilm. PAO1 + ETB-1 represents the 3-day *Pseudomonas aeruginosa* PAO1 and clinical *S*. *sanguinis* strain co-cultured biofilm. *Indicates a significant difference in the expression of *lasI*, *lasR*, *rhlI* and *rhlR* between the PAO1 and the PAO1 + ETB-1 group (*P* < 0.05). (**E**) IL-8 expression in different groups after stimulation with BCM. The x-axis indicates the 1 hour, 3 hour, 6 hour, 9 hour, 12 hour and 24 hour incubation of BEAS-2B cells with biofilm conditioned medium (BCM) of different groups. PAO1 represents BCM of 3-day *Pseudomonas aeruginosa* PAO1 biofilm. PAO1 + ETB-1 represents BCM of 3-day *Pseudomonas aeruginosa* PAO1 and the clinical *S*. *sanguinis* strain co-cultured biofilm. ETB-1 represents BCM of the 3-day clinical *S*. *sanguinis* strain biofilm. Control represents the brain heart infusion medium applied in the study of biofilm culture. *Indicates a significant difference in IL-8 expression between the PAO1 group and the PAO1 + ETB-1 group (*P* < 0.05).

To gain insight into the role of the clinical *S*. *sanguinis* strain in the development of VAP, we investigated its capacity for biofilm formation. Crystal violet assays (Fig. 5A), plate counts (Fig. 5B), and confocal laser scanning micrographs (Fig. 5C) all showed that the clinical *S*. *sanguinis* strain did not form a significant biofilm. *Pseudomonas aeruginosa* PAO1, which was widely distributed in our study samples, was able to form biofilm. We further assessed whether the clinical *S*. *sanguinis* isolate could affect the biofilm formation of PAO1 in the *in vitro* co-culture model of PAO1 and *S*. *sanguinis* isolate. When co-cultured with the *S*. *sanguinis* isolate, the PAO1 biofilm was significantly thicker (33.16 ± 1.12 μm vs. 21.50 ± 1.33 μm) (Fig. 5C), cell viability of the biofilm was higher (Fig. 5B) and biofilm biomass (Fig. 5A) was larger than that of mono-PAO1 biofilms (*P* < 0.05). Quorum sensing (QS) of *Pseudomonas aeruginosa* is associated with biofilm formation^19^. To further explore the possible mechanisms for the enhanced biofilm mass with the concurrent clinical *S*. *sanguinis* isolate, we investigated gene expression of the four critical QS genes, *lasI*, *rhlI*, *lasR*, and *rhlR*. We found that all four investigated genes were significantly upregulated in the *S*. *sanguinis* and PAO1 co-cultured biofilms compared to the mono-PAO1 biofilm (*P* < 0.05, Fig. 6A,B,C and D).

### IL-8 secretion in human lung/bronchus epithelia induced by BCM

After exposure to potential pathogens, airway epithelia cells produce IL-8 to initiate migration and activation of neutrophils and leukocytes against infection. Because IL-8 is critical in innate, and the thereafter, adaptive immune responses, its release by airway epithelia induced by the biofilm conditioned medium (BCM) was also investigated. The co-cultured *S*. *sanguinis* and PAO1 BCM induced a lower level of IL-8 compared with PAO1 BCM after intubation with human lung/bronchus epithelia BESA-2B cells for three hours (*P* < 0.05, Fig. 6E).

## Discussion

Biofilm formation on ETTs is a universal phenomenon and provides a bacterial reservoir for VAP among mechanically ventilated patients. To our knowledge, this is the first study to characterize complex microbial communities in ETT biofilms among intubated neonates based on next generation sequencing. Microbial investigations based on next generation sequencing have broadened our ability to identify the complex microbial world. Our study showed that this non-cultural approach can be used to characterize microflora in ETT biofilms. The richness and diversity of ETT biofilm flora have been underestimated by the conventional culture-based approach. Our results revealed a far more complex microflora in ETT biofilms from intubated neonates compared to the previous culture-based study. Additionally, we found for the first time that patients with different underlying diseases before intubation had distinct microbial signatures in their ETT biofilms. This finding suggests the importance of well-categorized cases based on underlying diseases to characterize the ETT biofilm flora, particularly to investigate its association with outcome. Therefore, the diversity of pathogens involved in VAP is far more complex than the current literature suggests.

Previous studies have reported that *Pseudomona*s *sp*. and *Enterobacter sp*. were common pathogens that caused VAP^20, 21^, and we found that the two genera were overabundant in ETT biofilms in our study samples. This finding validates the fact that biofilms serve as the source of pathogens for VAP. However, the two genera were also identified in the ETT biofilms of patients without VAP, suggesting that the colonization of *Pseudomonas sp*. and *Enterobacter sp*. in ETT biofilms is necessary but not enough to cause VAP. The prevalence of *Pseudomonas sp*. and *Enterobacter sp*. in ETT biofilms in our study samples is alarming, and strategies are urgently needed to minimize or eliminate their colonization among intubated neonates to reduce nosocomial infections in NICUs.

Oral bacterial species were considered to be the potential reservoir of pathogens involved in VAP^22^. Recent clinical trials have also shown that VAP incidence could be reduced by improving oral hygiene^23, 24^. In the current study, the abundance of Streptococci in ETT biofilms was significantly related to the onset of VAP, and Streptococci were reported to be the dominant members of normal flora in the oral cavity among infants^25^. This suggests an association between the oral flora in ETT biofilms and VAP. Previous non-cultural based studies among intubated adults also demonstrated the existence of oral flora in ETT biofilms, yet their role in VAP has not been addressed^16–18^. Our finding provides the first epidemiologic evidence of the role of Streptococci in ETT biofilms in VAP and therefore highlights the usefulness of addressing these commensal oral flora to prevent VAP among neonates.

The present study showed that the clinical *S*. *sanguinis* strain isolated from ETT biofilms could enhance the biofilm formation and viability of the common VAP pathogen, PAO1, *in vitro*. As one of the commensal oral species, *S*. *sanguinis* is pathogenic when entering the bloodstream to cause bacterial endocarditis or in some immune-comprised patients^26^. Multiple bacterial species in the microbiota are responsible for diseases rather than a single organism^27^. Bacterial members of the microbiota may interact with each other by competition and coordination^28^. A well-documented mechanism for the interactions among bacteria is cell-cell communication via QS cross talk. The QS system has been identified to regulate virulence factor expression, biofilm formation, and antibiotic production, all of which are associated with the pathogenesis of bacteria^19^. The communication is conducted via chemical signal molecules termed autoinducers (AI), which are produced and released by QS bacteria^29^. Genes involved in the QS system participate in the production of these AIs. For *Pseudomonas aeruginosa*, N-acyl homoserine lactone (AHL) is as an important AI as AI-1, which includes N-3-oxo-dodecanoyl-homoserine lactone (3O-C12-HSL) and N-butanoyl-homoserine lactone (C4-HSL). The QS Genes *lasI* and *rhlI* encode 3O-C12-HSL synthetase and C4-HSL synthetase, respectively. In addition, the *las* and *rhl* systems also consist of the transcriptional activator proteins *lasR* and *rhlR*. We found that the clinical *S*. *sanguinis* strain significantly upregulated expression of *lasI*, *rhlI*, *lasR* and *rhlR* of *Pseudomonas aeruginosa* PAO1 in the study. This finding might be a reason for the increased biofilm formation of PAO1 within the *S*. *sanguinis* isolate.

In addition, the co-culture *S*. *sanguinis* and PAO biofilm BCM induced a lower level of IL-8 release in airway epithelia cells than the mono-PAO biofilm *in vitro*. IL-8 can promote neutrophil recruitment and influence circulating leukocyte populations *in vivo* ^30^. Therefore, the IL-8 disparity *in vitro* in our study agrees with our finding of lower WBC counts among patients with *Streptococcus sp*. present in ETT biofilms. These findings imply that *Streptococcus sp*. in biofilms might alleviate the host immune response to putative pathogens, such as *Pseudomonas aeruginosa*. An impaired immune response results in the persistence of pathogens and infection^31^. It has been recently proposed that 3O-C12-HSL, mentioned earlier, could impair the function of nuclear factor-κB (NF-κB) and lead to the repression of NF-κB-responsive genes encoding inflammation cytokines, including IL-8^31^. Thus, the alleviated immune response with *S*. *sanguinis* might partly be a result of the increased gene expression of *lasI*, which encodes 3O-C12-HSL synthetase.

This study has several limitations. First, the sample size in the present study is relatively small, which may result in inadequate statistical power. However, we provide a preliminary insight into the microflora in ETT biofilms among intubated neonates, especially in view of the total lack of this type of data among intubated neonates in NICUs in China and other parts of the world. Antimicrobial therapy might be one of the confounding factors influencing microbial communities. It has been confirmed that biofilm formation was not associated with systemic antibiotics among patients with mechanical ventilation^11^. However, the fact that various types of antibiotics were administered according to the patient condition in the present study made it impossible to assess the potential role of antibiotics in profiling the ET biofilm microflora. The reads identified as members of Streptococci have not been assigned at the species level due to the limited resolution of the 16S rRNA gene. The other limitation is that we did not have sequential ETT samples during intubation for every intubated neonate, so the dynamics of the microbiome during intubation for an individual patient has not been revealed. However, sequential sampling for intubated neonates is not allowed due to the concern for patient safety, and re-intubation was also reported to be a risk for VAP^32^.

## Conclusions

This study extends the knowledgebase of microbiota in ETT biofilms from intubated neonates and provides new insight into the pathogenesis of VAP by suggesting that *Streptococcus sp*. may evolve in the onset of VAP by interacting with other nosocomial pathogens and regulating host immune responses. More epidemiological studies with larger sample sizes from intubated neonates are warranted based on which strategies can be developed to address this important nosocomial disease in NICUs.

## Methods

### Ethics statement

All experiments were performed following the relevant guidelines and regulations of CHCMU. The study protocol was reviewed and approved by the Medical Ethics Committee of CHCMU. Informed consent was obtained from the parents or legal guardians of the study patients. De-identified data were used in the data analysis.

### Patient involvement and classification

Neonates who suffered from mechanical ventilation for more than 48 hours in CHCMU during the study period from January 31, 2014, to July 31, 2014, were considered in the study. Patients diagnosed with NRDS and pneumonia before mechanical ventilation during the study period were consecutively enrolled and classified into either the NRDS or pneumonia group. To ensure NRDS patients would have normal oral flora and a sterile lower respiratory tract prior to intubation, NRDS patients with the following conditions were excluded, as previously described^33^: (1) intrauterine infection; (2) diagnosed with concurrent infectious diseases prior to intubation; and (3) mothers with a history of infections or who had used antibiotics during the last month of pregnancy. VAP was considered according to the definition by the CDC for infants younger than 1 year, including radiological signs, clinical signs & symptoms and microbiological findings^34^, based on which all of the study patients in NRDS and pneumonia groups were further identified as VAP and non-VAP cases. Empirical antibiotics were applied in all the study patients according to patient condition as determined by clinicians (Tables 2 and 3). NRDS, pneumonia and VAP were diagnosed by at least three experienced clinicians according to patients’ symptoms and X-ray findings.

### Sample collection and procession

ETT samples were collected immediately after extubation. The distal 2 cm of the tubes were cut off, transferred to 5 ml of phosphate buffered saline (PBS), and processed using a Vortex and sonication to release biofilms as previously described^17^.

### PCR amplification and Illumina Miseq sequencing

Microbial DNA was extracted using the TIANamp Bacteria DNA Kit (Tiangen, China) from the PBS solution containing recovered biofilm following the manufactory’s instructions and stored at −20 °C for further use. The V3–V4 region of the bacteria 16S rRNA gene was amplified using the primers 338F (5′ACTCCTACGGGAGGCAGCA-3′) and 806R (5′GGACTACHVGGGTWTCTAAT-3′). A unique barcode was added to the primer for the identification of each sample. Amplification was initiated by initial denaturation at 95 °C for 2 min, followed by 25 cycles at 95 °C for 30 s, 55 °C for 30 s, 72 °C for 30 s, and a final extension at 72 °C for 5 min. The PCR products were confirmed by 2% agarose gel electrophoresis. The DNA in the target band was recovered using an AxyPrep DNA Gel Extraction Kit (Axygen Biosciences, Union City, CA, U.S.), and amplicon sequencing was performed on the Illumina MiSeq platforms at Majorbio Bio-Pharm Technology Co., Ltd (Shanghai, China). Complete data sets are submitted to the NCBI Short Read Archive under accession no. SRP057949.

### Processing of sequencing data

Pairs of reads of the original DNA fragments were quality-filtered using QIIME (version 1.17) and merged using FLASH^35^. Sequence reads were assigned to each sample according to the unique barcode of each sample. QIIME and the UPARSE pipeline were used to analyse the sequences, and sequences were assigned to the OTU with 97% similarity as previously described^36, 37^. One representative sequence per OTU was selected and assigned to certain taxonomic data using RDP classifier (version 2.2) and was identified in the RDP database (Release 11.1 http://rdp.cme.msu.edu/)^38^. To evaluate the coverage of sequencing, coverage coefficients for each sample were calculated using Mothur software (version v.1.30.1)^39^. The Ace and Chao indices were calculated by Mothur to assess the richness of the microbial communities, and the Shannon Wiener index and the Simpson’s index were to evaluate the community diversity. β diversity of microbial environments visualized by PCoA and Adonis based on Bray-Curtis distances were performed in R (http://www.r-project.org/). The histogram of microbial communities for each sample was generated based on the relative abundance of each OTU. Bacterial species with a relative ratio of more than 1% were considered to be present in the sample.

### Isolation and identification of *Streptococcus sp*. strains

According to our preliminary data, *Streptococcus sp*. OTU was overabundant in ETT biofilms of VAP patients. To investigate the existence of *S*. *pneumonia*, the common causative agent of pneumonia, the *S*. *pneumonia* specific PCR was performed as previously described^40^. The released biofilm cell suspensions were inoculated onto Columbia blood agar plates and cultured for 48 h at 37 °C. Bacterial isolates were identified as *Streptococcus sp*. according to haemolysis, gram positive reaction, coccus morphology arranged in chains, and negative catalase test results^41^. Bacterial DNA was extracted as mentioned earlier. The concatenated sequences of the seven housekeeping genes were amplified and sequenced as previously described^42^. The phylogenetic tree was constructed via MEGA5 (http://www.megasoftware.net/). Strains were assigned to species on the basis of their distance from species type strains^41, 42^.

### Biofilm culture

Bacterial biofilms were cultured in brain heart infusion (BHI) (Nissui, China) medium at 37 °C 5% CO_2_ for three days^43^. BCM was collected as previously described^44^. Plate count was conducted to evaluate the viability of bacteria in biofilms. Crystal violet assay was performed to compare the biofilm biomass as described^43^. Biofilms were stained with a LIVE/DEAD BacLight kit (Invitrogen, U.S.) and observed using confocal laser scanning microscopy (CLSM). The CLSM image was analysed using ImageJ (http://imagej.nih.gov/ij/)^43^. Gene expressions of the critical QS genes including *lasI*, *rhlI*, *lasR*, and *rhlR* were investigated via Real-time PCR as previously described^45^.

### IL-8 detection

Human lung/bronchus epithelia (BEAS-2B, ATCC-9609) were cultured in DMEM: F12 medium (Invitrogen, U.S.) with 10% fetal bovine serum (Hyclone, U.S.), 100 U/ml penicillin, and 100 mg/ml streptomycin at 37 °C in 5% CO_2_. The supernatants were collected after stimulation with BCM for 1 h, 3 h, 6 h, 9 h, 12 h and 24 h. The secretion of IL-8 was quantified using enzyme-linked immunosorbent assay (ELISA) following the manufacturer’s instructions (4A Biotech, China).

### Statistical analysis

Continuous variables were expressed as the mean ± SEM or median (P25–P75) according to whether they followed a normal distribution. Dichotomous variables were presented as number (percentage). The comparisons among continuous data were analysed by Student’s t test, adjusted t-test, ANOVA, and Mann-Whitney U test as appropriate. The Chi-square test or Fisher’s exact test was used for comparisons among dichotomous variables. The association between OTU count and VAP was investigated using the Spearman test. All the statistical analyses were performed using SPSS (version 17.0). *P* value less than 0.05 was considered to be statistically significant.
